# Analysis of Methods for Determining Shallow Waterbody Depths Based on Images Taken by Unmanned Aerial Vehicles

**DOI:** 10.3390/s22051844

**Published:** 2022-02-25

**Authors:** Mariusz Specht, Marta Wiśniewska, Andrzej Stateczny, Cezary Specht, Bartosz Szostak, Oktawia Lewicka, Marcin Stateczny, Szymon Widźgowski, Armin Halicki

**Affiliations:** 1 Marine Technology Ltd., Wiktora Roszczynialskiego 4-6, 81-521 Gdynia, Poland; m.specht@marinetechnology.pl (M.S.); m.wisniewska@marinetechnology.pl (M.W.); m.stateczny@marinetechnology.pl (M.S.); s.widzgowski@marinetechnology.pl (S.W.); a.halicki@marinetechnology.pl (A.H.); 2 Department of Geodesy, Gdańsk University of Technology, Gabriela Narutowicza 11-12, 80-233 Gdańsk, Poland; bartosz.szostak@pg.edu.pl; 3 Department of Geodesy and Oceanography, Gdynia Maritime University, Morska 81-87, 81-225 Gdynia, Poland; c.specht@wn.umg.edu.pl (C.S.); o.lewicka@wn.umg.edu.pl (O.L.)

**Keywords:** bathymetric measurements, shallow waterbody, photogrammetric image, Unmanned Aerial Vehicle (UAV), Unmanned Aerial System (UAS)

## Abstract

Hydrographic surveys enable the acquisition and processing of bathymetric data, which after being plotted onto nautical charts, can help to ensure safety of navigation, monitor changes in the coastal zone, and assess hydro-engineering structure conditions. This study involves the measurement of waterbody depth, identification of the seabed shape and geomorphology, the coastline course, and the location of underwater obstacles. Hydroacoustic systems mounted on vessels are commonly used in bathymetric measurements. However, there is also an increasing use of Unmanned Aerial Vehicles (UAV) that can employ sensors such as LiDAR (Light Detection And Ranging) or cameras previously not applied in hydrography. Current systems based on photogrammetric and remote sensing methods enable the determination of shallow waterbody depth with no human intervention and, thus, significantly reduce the duration of measurements, especially when surveying large waterbodies. The aim of this publication is to present and compare methods for determining shallow waterbody depths based on an analysis of images taken by UAVs. The perspective demonstrates that photogrammetric techniques based on the SfM (Structure-from-Motion) and MVS (Multi-View Stereo) method allow high accuracies of depth measurements to be obtained. Errors due to the phenomenon of water-wave refraction remain the main limitation of these techniques. It was also proven that image processing based on the SfM-MVS method can be effectively combined with other measurement methods that enable the experimental determination of the parameters of signal propagation in water. The publication also points out that the Lyzenga, Satellite-Derived Bathymetry (SDB), and Stumpf methods allow satisfactory depth measurement results to be obtained. However, they require further testing, as do methods using the optical wave propagation properties.

## 1. Introduction

Bathymetric works that enable the measurement of shallow waterbody depths is necessary for acquiring geospatial information describing the marine environment. Information on a waterbody’s depth has a direct effect on navigational safety and efficiency, coastal zone management, the process of hydro-engineering structure designing and monitoring, and a range of other types of human activity at sea [1,2].

The most popular devices for waterbody depth measurement include Single Beam Echo Sounders (SBES) and MultiBeam EchoSounders (MBES). Even though SBES echo sounders continue to be the most commonly used bathymetric systems worldwide, it is the MBES echo sounders that, thanks to their large swath width, are able to ensure complete coverage of the seabed with depth data and enable the performance of such work in a relatively shorter period of time [3].

However, over the last decade, the increasing impact of depth measurement methods providing alternatives to hydroacoustic methods has been observed. These methods include photogrammetric and remote sensing techniques that enable the acquisition of depth data to generate three-dimensional seabed models. Of all the measurement methods other than hydroacoustic, Airborne Laser Scanning (ALS) [4] deserves a special mention. It is similar in terms of the swath width to the surveys carried out by the MBES [5].

Other current alternative (and very quickly developing) methods for acquiring depth data include satellite bathymetry [6] and multispectral imaging [7] based on the Satellite-Derived Bathymetry (SDB) method. It should be emphasised that the use of satellite images is less costly than any other bathymetric measurements, especially compared to Light Detection And Ranging (LiDAR) systems. However, this method fails to ensure the required accuracy of depth measurements [8].

Another remote sensing technique that enables the performance of bathymetric measurements is the Airborne LiDAR Bathymetry (ALB), also referred to as the Airborne Laser Hydrography (ALH), which has been evolving rapidly in recent years [9]. Its operation is based on the application of green lasers, and the depth value itself is determined through the knowledge of the two-directional course of a laser beam between the water surface and the reflections from the seabed located underneath [10]. Systems such as the ALB/ALH are mounted on board aircraft or helicopters, but due to progressive equipment miniaturisation, sensors of this type can be mounted on Unmanned Aerial Vehicles (UAV) [11,12].

Currently, the use of UAVs in hydrography has enabled the acquisition of high-resolution geospatial data and ensured considerable precision in determining their coordinates. The development of unmanned aerial vehicles is one of the challenges for the aviation industry, and they offer alternative measurement solutions, e.g., for terrain modelling, monitoring changes (including in the coastal zone), and environmental protection [13]. UAVs are commonly used when developing geospatial models of terrestrial areas, but their use in bathymetric measurements is accompanied by errors due to the phenomenon of water-wave refraction. Hence, in order to acquire images of an area, camera calibration and correct stabilisation are required. These are performed based on appropriately designed and measured Ground Control Points (GCP), also referred to as photopoints [14], which ensure the so-called direct georeferencing [15]. These points must be visible on at least several images, which, when working on waterbodies, limits the possibility of placing them on land areas (with the exception of waterbodies with high purity and clarity levels). Nevertheless, the use of unmanned platforms in bathymetric works has a number of advantages, i.e., a shorter measurement time, mobility and lower costs compared to traditional methods using hydroacoustic systems.

This study also presents the current methods for determining shallow waterbody depths based on an analysis of images taken by UAVs. The selection of the appropriate method is crucial, as it is planned to acquire accurate and reliable bathymetric data in the coastal zone. The accuracy of the determination of the waterbody coordinates must meet the requirements imposed for the order of hydrographic surveys according to the International Hydrographic Organization (IHO) standard S-44 [16]. In view of the above-mentioned advantages of work using unmanned aerial vehicles, it is considered necessary to review modern methods for determining shallow waterbody depths based on different techniques related to both image processing and further work on acquired hydrographic products. These include the Digital Elevation Model (DEM), Digital Surface Model (DSM) and orthophotomaps, in order to achieve the highest possible accuracy of depth measurements.

The main aim of this publication is to compare modern methods for determining shallow waterbody depths. It will provide information useful for selection of appropriate method. It is achieved by:

1. Summarise the most promising methods for determining the waterbody depth. This literature review can provide a structured base of knowledge on the modern task of determining the waterbody depth based on images taken by an UAV. This study provides the reader with the basis for a detailed analysis of individually selected solutions;
2. Analyse the literature, which enables the extraction of basic information from individual studies. The specification of waterbody descriptions, designed control points, as well as the hardware and software used may allow the selected method to be adjusted to the technical parameters of particular survey and research equipment. Moreover, the method for verifying the obtained measurement results in-situ;
3. Compare and define the advantages and disadvantages of each particular method through an assessment of the results obtained by the method authors. With reference to the above, two points, differences and similarities were also defined. The values of individual errors were compared, thus enabling the selection of the most promising technique.

The remainder of this article is organised as follows: the “Materials and methods” section presents the concept of operation of five methods for determining waterbody depths, with their descriptions taking account of the data recording, location and mission preparation. The “Results” section summarises and comparatively analyses selected methods in terms of the obtained measurement results. The “Discussion and conclusions” section presents the formulated advantages and disadvantages of each of the analysed methods.

## 2. Materials and Methods

Many of the methods described in this chapter are based on the Structure-from-Motion (SfM) technique. SfM [17] is a technique that has been actively tested in recent years [18], and its task is to provide three-dimensional scenes using a series of temporal RGB images and georeferencing information. It also provides information on the internal and external camera orientation at the time of acquiring each image by using automatic algorithms for estimating its location. This results in a model that enables the determination of how individual 3D coordinates are projected on the images from the camera [19,20]. When working using the SfM technique, it is important to logically plan the GCP point network. A properly aligned network improves the extraction of metric data and data transformation to an actual coordinate system [21]. The Multi-View Stereo (MVS) [22] is a general term for a group of stereo photography techniques that involve taking two images of an object from different viewpoints [23].

**Figure 1:**
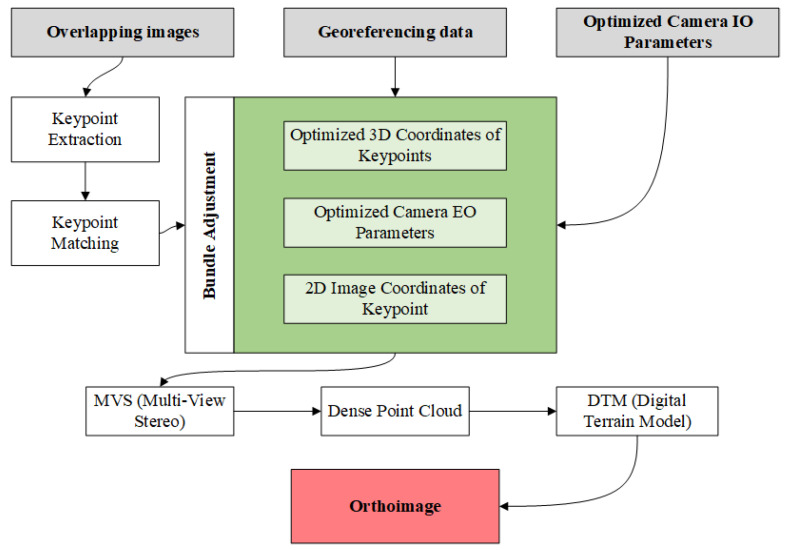
Diagram of the Structure-from-Motion-Multi-View Stereo (SfM-MVS) algorithm’s operation, whose final result is an orthoimage based on [27].

An essential step when working with the SfM-MVS algorithms is the design and measurement of photopoints [24], which can be performed by using the tachymetric measurement method based on the local control network, or with a Global Navigation Satellite System (GNSS) receiver [25]. Figure 1 shows the commonly used operating process based on the SfM-MVS algorithm that results in an orthoimage. It should be mentioned, however, that almost every reference in the literature identifies the phenomenon of water-wave refraction as a threat when working with SfM-MVS algorithms. The effect of light refraction at the air/water interface must be eliminated when processing photogrammetric data [26].

The use of the SfM-MVS technique to develop a DEM has been presented, e.g., in [28] for developing bathymetric and topographic reconstructions. The reconstruction of coastal water was performed by using the Agisoft Metashape (St. Petersburg, Russia) software that contains SfM-MVS algorithms. As in the case of [29], the phenomenon of water-wave refraction was recognised as a threat to the application of the above-mentioned technique. The experiment described by the authors was aimed at comparing DEM reconstructions, or rather its efficiency with an increasing water level, i.e., changing depth. This was possible by selecting the site of a coral reef on Fuvahmulah Island (Maldives) at low tide and at rising tide. The study area covered a sector with 12 control points which, at a further stage, were crucial in the SfM-MVS process in georeferencing and point cloud scaling. For the recording of aerial images, a DJI Phantom 4 Pro V1.0 (Shenzhen, China) with a nadir-viewing camera was used. The photogrammetric measurements covered an area with a length of 140–150 m along the shore, a width of 70–80 m across the shore, and a height of approximately 25 m. Information about the measurement site are presented in Table 1. In order to eliminate errors due to water-wave refraction, the authors measured GCP points under the water surface and applied an algorithm written in the Python (Beaverton, OR, USA) programming language (script: PY_FM_DEPTH). The accuracy of depth measurement decreased as the depth increased, i.e., above 1 m. Above this value, the highest accuracies were obtained using GCP points and with no correction applied to eliminate the effects of the phenomenon of water-wave refraction. These, however, were not satisfactory.

**Figure 2:**
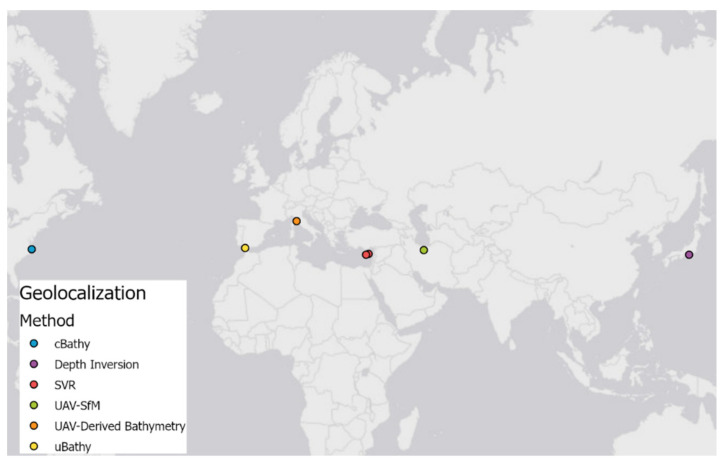
Map presenting the location of each measurement site according to the method used to determine the waterbody depth.

In the following subsections, there are presented methods that use SfM data for shallow waterbody depth determination. Table 2 lists the names of algorithms along with information about measurement locations (Figure 2) and equipment used during surveys.

### 2.1. Depth Determination Based on the cBathy Method

A less popular, yet interesting, solution for determining depths is the use of the cBathy algorithm. The concept of the algorithm presented by [30] is based on observations of surface wave movements over long time series. The estimation of bathymetry is possible by determining the relation between the wave velocity and the depth. The algorithm’s operation can be divided into three basic stages:

1. Analyses dependent on the candidate frequency, radial wave number, equivalent depth and the wave angle. The analyses are based on a Fourier transformation. This stage involves such operations as the determination of optimum wave numbers and their directions, and the use of the Levenberg-Marquardt algorithm. Once this step is completed, a set of wave numbers and depths dependent on a particular frequency is obtained;
2. The estimation of a single, best depth value, based on the dispersion relation. The previous step provided a set of wave numbers and depths, while this stage enables the determination of a single depth based on the set obtained. To this end, a Hanning filter is used;
3. Estimation of the average running depth, aimed at obtaining a moving average value to eliminate problems related to the emergence of gaps in data, e.g., resulting from temporary loss of view during the course. At this stage, the value of a Kalman filter enhancement is calculated [31].

A study by [30] describes the course of the tests conducted in order to validate the cBathy algorithm. These were conducted at two locations: on a waterbody located at the Field Research Facility (FRF) in Duck and on the Agate Beach coastline (USA). As regards the first site, the study was based on 16 sets of measurement data recorded in the years 2009–2011, while for Agate Beach, only 1 set was involved. However, the testing under this study was not conducted using an UAV or on an area other than that covering ocean waters. Nevertheless, the study provides a number of mathematical formulas that explain the algorithm step-by-step.

Based on the cBathy algorithm, bathymetric measurements were conducted using the Unmanned Aerial System (UAS) on two waterbodies: the above-mentioned FRF in Duck, and on the Virginia Beach coastline (USA) [32]. In the process of image acquisition, a 3D Robotics X8+ platform (Berkeley, CA, USA), 4 GoPro Hero 4 Black devices (San Mateo, CA, USA), a Teensy 3.2 microcontroller (Cambridge, UK), an Adafruit Ultimate GPS receiver (New York, NY, USA) and an openLOG data recorder (Niwot, CO, USA) were used. The microcontroller, GPS receiver and data recorder enabled the acquisition of images from four GoPro devices at the same time, with a refresh rate of 30 Hz, i.e., 30 fps. The use of GoPro cameras enabled the acquisition of video images, as these devices are characterised by a high value of the Field Of View (FOV) parameter that is responsible for a wide field of view. In order to synchronise the image between GoPro devices, it was decided to use sound recorded along with the image. Moreover, the National Marine Electronics Association (NMEA) communications, recorded by the microcontroller, enabled the synchronisation of time between the cameras and the GPS receiver. The synchronisation was done in post-processing.

On the basis of the GNSS/Inertial Navigation System (INS), the GCP points were measured. The images were processed using the SfM-MVS technique in the Agisoft Photoscan software (St. Petersburg, Russia). In this way, a point cloud was obtained, which was later subjected to linear interpolation based on triangulation in the MATLAB software (Natick, MA, USA). Consequently, a DSM model was obtained. Moreover, an orthophotomap was generated in the MATLAB program. Prior to the application of the cBathy algorithm, the images were converted into grayscale. The image contrast was enhanced based on the Contrast Limited Adaptive Histogram Equalization (CLAHE) algorithm, and the blank pixel values were replaced with time-averaged intensity values for particular pixels. The use of the cBathy algorithm parameters proposed in [30] enabled the reduction of a number of points whose depth measurement error was greater than 1 m. However, no Kalman filtration, i.e., an element of the original cBathy algorithm, was used. The end result was a new model called “Topo-Bathy DSM”, generated based on a dense point cloud.

The photogrammetric measurement results obtained using the UAS system were compared with the results acquired from soundings conducted by a vessel equipped with an echo sounder and a Global Positioning System (GPS) Real Time Kinematic (RTK) receiver. The Root Mean Square Error (RMSE) range for depth measurements was 0.17–0.34 m. The greatest differences in the depth values could be observed near the pier and in close proximity to the coastline.

### 2.2. Depth Determination Based on the Depth Inversion Method

The article [33] presented the Depth Inversion method, which enables the determination of the waterbody depth based on wave propagation resulting from the combination of the wind force, its duration, and the gravitational force which is detected from video images. The authors believe it is possible through the conversion of original video images into orthogonal images based on photopoints and the cross-correlation analysis of pixel intensity signals. This analysis allows the wave-celerity vector field to be defined and, thus, the wave period to be determined. The estimated wave parametres can be converted into values representing water depth by using the dispersion relation from linear wave theory. In order to calculate the depth values, the following parameters are needed: wave frequency, wave velocity and gravitational acceleration. It is more advantageous to use long-period waves, as the wavelength affects the strength of the dependence of the wave velocity on water depth.

For testing purposes of the proposed method, recordings were made using an UAV in ocean waters located in Suruga Bay (Japan), at the mouth of the Oi River. For the video observation, a DJI Phantom 4 unmanned aerial vehicle (Shenzhen, China) with a mounted camera with a resolution of 4K and a refresh rate of 29.97 Hz, whose horizontal angle was set diagonally to the coastline. As with each of the methods discussed, photopoints were designed and measured on land, and the image template fitting algorithm (finding a similar template in a source image based on the base template, performed in order to compare) enabled the detection of image coordinates in each video frame, which allowed orthogonal video images to be generated. The recording was also made using the hydroacoustic system that was applied to determine the depth Root Mean Square (RMS).

The obtained depths were compared to the results obtained using the cBathy algorithm (discussed in the Section 2.1). The process of depth value determination in the cBathy method is based on the smoothing using the Kalman filter, while the Depth Inversion method applies the Gauss filter (5 × 5). Therefore, the authors stated that the comparison of results obtained from both methods failed to provide sufficient information to determine which of the methods was more accurate. In the area with breakwaters located along the coastline, both methods prevented the determination of depths more than 5 m, which can be associated with strong waves, or too few GCP points. Based on the tests, it could have been theoretically assumed that the Depth Inversion method can obtain depth measurement accuracies higher than those acquired using the cBathy algorithm. This was because the depth RMS error for the Depth Inversion method ranged from 0.33 to 0.52 m, while it ranged from 0.38 to 0.65 m for the cBathy algorithm.

### 2.3. Depth Determination Based on the SVR Method

The authors [29] assume that the images taken using UAVs can provide an alternative shallow seabed mapping method, provided that the Support Vector Regression (SVR) model is applied. SVR [34] is an algorithm that operates based on computation of the linear regression model in a multidimensional feature space [35]. The publication presents a proposal to eliminate the problem of water-wave refraction based on machine learning tools. The model is intended to enable a more accurate depth determination of point clouds acquired using the SfM-MVS technique.

The SVR method was verified using the data originating from two areas, i.e., Agia Napa and Amathouda (Cyprus). Photogrammetric surveys were conducted using a Swinglet CAM UAV (Raleigh, NC, USA) with a Canon IXUS 220 HS camera (Tokyo, Japan) mounted on it. Table 3 presents camera specification used during measurements, flight height and obtained Ground Sampling Distance (GSD). The LiDAR data were measured using a Leica HawkEye III bathymetric laser system (Wetzlar, Germany). During data recording, the average UAV flight altitude was 209 m (Agia Napa) and 103 m (Amathouda). Obviously, the GCP points were also designed. At the next stage of work, the data were pre-processed, and the number of image-based point clouds was reduced by half, as the LiDAR data were not sufficiently dense, and in order to apply machine learning, information on depth for each point originating from LiDAR and the image was required. In this step, image-based points were eliminated whose height was greater than or equal to 0 m and the elevation was recorded by the LiDAR.

As previously mentioned, the threat arising from the phenomenon of water-wave refraction can be eliminated, e.g., based on the SVR—a supervised algorithm used to predict discrete values. This regression model tries to minimise, in its computations, the error between the predicted value and the actual value [36]. In the case of this method, a SVR model that uses the implementation of the scikit-learn module (Python) was applied. The module comprises a number of machine learning algorithms [37]. Training is performed using the information on the individual points’ depths. The data were acquired using point clouds developed based on the images recorded by the camera and LiDAR. The training, testing and validation stages were based on six training sets originating from the above-mentioned test areas, i.e., Agia Napa and Amathouda, which were divided. The first four approaches were aimed to fit the linear SVR model and to predict the correct depth based on individual sets (the datasets were not used in 100%). The fifth approach already included 100% of the datasets and their combination. The final (sixth) approach enabled the generation of a virtual dataset. The point clouds obtained using the SfM-MVS technique were compared with the point clouds developed in the 3D Cloud Compare software used for editing and processing point clouds, which uses the Multiscale Model to Model Cloud Comparison (M3C2) algorithm whose operation is based on the measurement of the distance along the normal vector. The normal vector is estimated based on the neighbourhood of each point [38]. The determination coefficient (R^2^) was used to assess the fit of point clouds.

Based on the positive results of twelve out of thirteen different tests, it can be assumed that the results for the method using both the SfM-MVS and SVR techniques were shown to be very promising in terms of the depth measurement accuracies achieved. Moreover, this method is intended to be universal, i.e., independent of the UAV model and the equipment mounted on it. It was also proven that the training of a model from an area can provide results that are applicable on other, differing waterbodies. Moreover, several threats were defined, including the limitation of the method in areas where the seabed is characterised by the occurrence of dense sea grass or the lack of synchronicity between the point clouds obtained from the LiDAR and from the image. The authors of the method stress that work based on this model allows the accuracy requirements imposed on the IHO special order to be met [29].

### 2.4. Depth Determination Based on the UAV-Derived Bathymetry Method

An interesting solution [39] for bathymetric measurements using unmanned aerial vehicles is the UAV-Derived Bathymetry (UDB) method developed based on the SDB method [40], which uses algorithms that operate based on multi-spectral images [41], which are able to ensure a spectral resolution higher than RGB images by recording image data in specific electromagnetic spectrum range [42]. The depth values obtained following the application of the novel UDB method were compared with the results obtained from satellite images and SDB (using the Stumpf algorithm) and on the results obtained from traditional bathymetric measurements using MBES and SBES echo sounders.

The study was conducted in an area located on the Tyrrhenian Sea coast (Italy), along which an irregularly shaped coastline runs. In order to acquire measurement data, a HexaCopter UAV, equipped with a MAIA multi-spectral camera (Russi, Italy) comprising 8 multi-spectral sensors and a RGB sensor was used. The pre-processing of the image was performed using camera-dedicated MAIA software (Russi, Italy), which enables the correction of raw images and the generation of a multi-spectral image. Disturbances on the water surface due to sunny weather caused further disturbances that had to be reduced by using Hedley’s method, i.e., an upgraded technique for eliminating the “reflection” from remote sensing images, based on the use of near-infrared region [43]. The same algorithm was applied in [8] using satellite images.

The problem of image georeferencing due to the lack of possibility of using the SfM technique in the UDB method was solved by calculating planar deformation based on the knowledge of such parameters as location (based on GPS receiver indications), image size, course value and the angles recorded during flight. With reference to the SDB, an attempt was made to determine the waterbody depth using the Lyzenga [44] and Stumpf algorithms [45]. Both models could have been converted in the ENVI 5 software (Melbourne, Australia), but when working with the Lyzenga algorithm, the ArcGIS program (Redlands, CA, USA) was used as well. Tests were conducted for 3 sets of the seabed control points uniformly distributed over the study area. Each set contained a different number of points (50, 200 and 500).

The authors believe that the technology using UAVs in bathymetric measurements may provide an alternative to research carried out by traditional methods using MBES and SBES echo sounders, yet only for waterbodies that do not require high accuracy. The results obtained by the novel UDB method were promising and reliable, as compared to the commonly known method based on satellite images. The depth values acquired using the Stumpf algorithm were similar to those calculated using the Lyzenga algorithm. It is worth mentioning that the Stumpf method is a simpler one, because it does not require as many regression methods, as is the case with the second algorithm.

### 2.5. Depth Determination Based on the UAV-SfM Method

Another method based on the SfM technique is the UAV-SfM method described by [46]. The waterbody selected by the authors was the Alarm River in Iran, because it is characterised by diverse hydrological and morphological conditions. The data required for study performance were collected using a Spreading Wings S1000 UAV (Shenzhen, China) and a Canon 5D Mark III camera (Tokyo, Japan) mounted on it, with a flight altitude of 32 m. This method also measured the GCP points and the data processing using the SfM technique was performed in the Agisoft PhotoScan Pro software (St. Petersburg, Russia). A total of nearly 9450 RTK GNSS points were collected. In order to use the algorithms that enable the correction of the errors due to the refraction phenomenon, the authors propose a solution involving the generation of a water surface model. The concept of constructing the water surface model was based on the extraction of water edges from individual products (DEM and the orthophotomap) that were results of work with the SfM. The construction of DEM was based on the use of the Triangulated Irregular Network (TIN) model [47]. The authors stressed that an important aspect of the work was the elimination of images of poor visual quality, i.e., blurred ones, even though the use of the SfM technique allows blurred images to be adjusted. Processing in the above-mentioned Agisoft PhotoScan Pro software using the SfM in order to obtain the DEM is possible through:

- image adjustment;
- photopoint importing;
- camera position optimisation;
- point cloud construction;
- network construction;
- texture construction.

The elevation of the seabed model, taking into account the phenomenon of water-wave refraction, could be determined by applying the appropriate correction algorithm [48]

(1) $$E_{RC}=E_{0}-0.34\left(E_{0}-WSE\right),$$

where:

*E_RC_*—DEM corrected elevation;
*E*_0_—DEM elevation;
*WSE*—water surface elevation.

It is stressed, however, that the algorithm can only be applied to a maximum depth of 0.7 m. It was also found that the greatest errors related to estimating elevation values were observed mainly near the shore or in areas with increased density of vegetation.

### 2.6. Depth Determination Based on the uBathy Method

The uBathy algorithm described in [49] refers to the previously discussed cBathy algorithm. However, it is based on the Principal Component Analysis (PCA) of the Hilbert transform as a function of time. The process is carried out on video images in order to determine the frequency and wave number for individual wave components. For each sub-video: the principal component analysis of the Hilbert transform of the grayscale frame intensity is performed, as well as the wave frequency (ω) and the space-varying wave number (k) are extracted for each of the main decomposition modes, and wherever possible. Both values (ω and k) should be obtained from the sequence of recorded video images. After completing the process for all sub-videos, the domains xy and a set of N pairs (ω_i_, k_i_), from which the water depth value (h) can be concluded while taking into account the dispersion relation, are obtained for each point. This method enables the estimation of waterbody bathymetry based on the propagation of electromagnetic waves that are recorded by a video monitoring system.

As part of a study by [50], aimed at learning about the effect of camera calibration and stabilisation on bathymetry estimation, a two-day measurement campaign was conducted on the urban Victoria Beach located on the south-western coast of Spain (the Atlantic Ocean). The depth values obtained from images provided by UAVs were compared with the results of bathymetric measurements acquired traditionally, i.e., using the SBES along with RTK-GPS positioning. A total of 34 photopoints were designed and measured (RTK-GPS), and distributed along the coastline. The study used a DJI Phantom 3 Pro unmanned aerial vehicle (Shenzhen, China) with a digital camera mounted on it. Based on projective geometry equations, it was possible to map the 3D coordinates of the real world. The authors of this publication stress that lens distortion must be taken into account in the camera calibration process and that both external and internal calibration of video frames is required as well. Prior to the application of the uBathy algorithm, all frames from the already calibrated videos had to be projected onto a plane, and the spatial domain was the intersection, in the two-dimensional plane (x, y), of the projections in the pixel domain. Carrying out this process allowed a grayscale, necessary in this process, to be obtained. The bathymetry estimation at each point was based on the previously mentioned process related to obtaining the wave frequency (ω) and the space-varying wave number (k), as well as based on the dispersion relation. Unfortunately, the depth estimation results contained gaps. Nevertheless, the results were promising, as the depth RMS error was approx. 0.4 m in relation to the depth values obtained using a SBES.

## 3. Results

**Figure 3:**
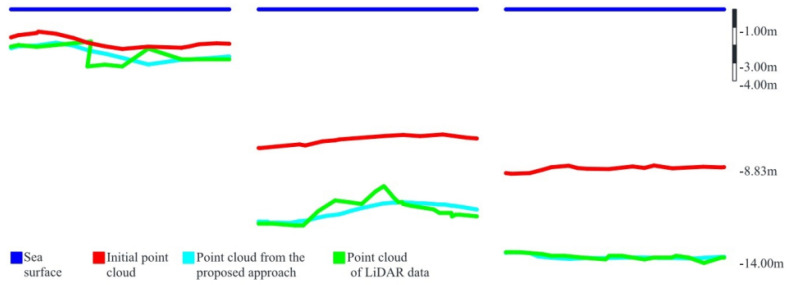
Data comparison between point cloud obtained from bathymetric Light Detection And Ranging (LiDAR) measurements (marked by green lines) and processed SfM point cloud (marked by red lines) by the Support Vector Regression (SVR) algorithm (marked by sky-blue lines). Water surface is also indicated by a blue line [29].

The results of bathymetric measurements, obtained using the methods presented in Section 2, were in almost every case compared with the results, shown in Figure 3, acquired using traditional methods and described by the authors as satisfactory.

For the cBathy, Depth Inversion, uBathy, and UDB algorithms, the depth RMSE values were provided and summarised in Table 4. The RMSE measure was selected because it is the most commonly applied criterion to assess the accuracy of the algorithms used for depth determination. Regarding the cBathy method, the range of depth RMSE value was developed based on the results obtained from two locations. The RMSE range for the Depth Inversion method was developed based on the results obtained from four flight passes that were performed on different days, i.e., under various weather conditions. Regarding the UDB method, the range of RMSE values was divided in relation to the depth, and the algorithms (Lyzenga and Stumpf) were applied. The RMSE range for the uBathy method was calculated based on the measurements originating from two films that were divided depending on the flight altitude. During the recording of video 1, the flight altitude was approx. 100 m, while it was approx. 50 m for video 2. The results for the uBathy algorithm were obtained by applying the Butterworth filter with a characteristic length of t_f_, i.e., the filtering characteristic times (0, 5, and 10 s).

Based on Table 4, it should be concluded that the depth accuracies obtained for the cBathy, Depth Inversion, and uBathy algorithms are similar. Where the UDB method was applied, a high depth accuracy was obtained for the range of 0–5 m. It should be noted, however, that this accuracy decreases as the depth increases. The highest depth accuracies (0.17–0.34 m) were obtained using the cBathy algorithm. The authors of the Depth Inversion method compared the results obtained based on their own assumption with the results acquired based on work using the cBathy algorithm. They demonstrated that the presented method could, in theory, obtain more accurate results. However, in order to confirm this thesis, more tests need to be carried out.

## 4. Discussion and Conclusions

Bathymetric measurements are often based on processing using the SfM, which is understandable in view of the advantage of this technique over traditional digital photogrammetric methods [51]. Chapter 2 also presents methods using other techniques. The depth RMSE values for individual methods can be compared with each other. It should be noted, however, that the study was carried out at sites differing in coastal morphology and under different weather conditions. Moreover, not all publications used the RMSE measure to evaluate the accuracy of the algorithms applied to determine the points’ depth. Each study described the effects as satisfactory and concluded that the results met the assumed minimum accuracy requirements.

The unquestionable advantage of the SVR method is the opportunity to apply the model to waterbodies other than the areas on which the training was conducted. The solution is based on machine learning tools. The authors can demonstrate high compliance between the results obtained by this method with the results acquired using the LiDAR system. It should be mentioned, however, that these values were not compared to the depths obtained by traditional depth measurement methods applied in hydrography. Hence, the results obtained may not be fully reliable.

The authors of the UAV-SfM method [46] used a number of already existing algorithms in their study, thus demonstrating their practical knowledge of the subject matter. The SfM processing with the use of the Agisoft PhotoScan software and the acquired hydrographic products are described briefly and clearly. Information on the description of the data recording stage and guidance on the evaluation of the recorded images may be particularly useful. However, detailed study results are lacking. It was further stressed that this method was only reliable for depths not exceeding 0.7 m.

As mentioned in Section 3, the highest depth accuracies (based exclusively on the RMSE measure) were obtained by applying the cBathy algorithm [30,32]. Data processing can be automated through the use of the Agisoft Photoscan software. The final result is affected by filtration, during which points with depths exceeding 1 m are eliminated. Another advantage of this algorithm is the use of Empirical Orthogonal Functions (EOF) to obtain an appropriate spatial resolution of the image. Gaps in the data can be filled using the Kalman filter, which enables the smoothing of depth data over time. All depth errors could have been mainly due to the environmental conditions, including the number of wave refractions, lighting, or the coastal relief (including the land cover). The final result could also be affected by camera synchronisation errors and camera lens distortion errors.

Based on Table 4, it should be concluded that the uBathy algorithm provides average depth measurement results compared to other methods. A significant disadvantage of this method is the opportunity to analyse the video two-dimensionally (as is the case for the cBathy algorithm) in order to obtain the period and length of the transverse wave. To eliminate errors depending on the waterbody and weather conditions, three different approaches to determining the wave number were developed. These approaches also differ in computational complexity. During the study carried out at Victoria Beach, based on the calculated depth values, the waterbody surface was measured, despite being incomplete due to gaps in the data. This was probably due to the phenomenon of water-wave refraction, too much insolation, and/or camera stabilisation problems.

Depth RMSE values similar to those obtained by applying the uBathy algorithm were acquired for the Depth Inversion method. The results presented in [33] suggest that this method performs well, provided that waves generated by storm winds are included in the observations. It is characterised by a rather high efficiency and low computational costs. The main disadvantage of the Depth Inversion method, however, is the fact that the depth accuracies vary significantly depending on the waving intensity. It is possible to correct this disadvantage by amplifying the signal of the above-mentioned mechanical waves. To this end, the camera settings must be adjusted accordingly. Nevertheless, a number of tests conducted under different weather conditions are still needed.

Very satisfactory depth measurement results were obtained using the UDB method, in particular when applying the Lyzenga model. The algorithm, based on the commonly used SDB method, allows a measurement accuracy higher than that of satellite bathymetry to be obtained. The application of the UDB algorithm also enables high efficiency of bathymetric works. It should be noted, however, that bathymetric measurements are more accurate at shallower depths, and it is shallow waterbodies that are the focus of this publication. The authors believe that the technology using UAVs in bathymetric measurements may provide an alternative to research carried out by traditional methods using MBES and SBES echo sounders, yet only for waterbodies that do not require high accuracy. The results obtained by the novel UDB method were promising and reliable, as compared to the commonly known method based on satellite images. The depth values acquired using the Stumpf algorithm were similar to those calculated using the Lyzenga algorithm. It is worth mentioning that the Stumpf method is a simpler one, since it does not require as many regression methods, as is the case with the second algorithm. The tool for determining the shallow waterbody depth requires further testing, which is the main disadvantage of this method. Not all limitations have already been recognised, e.g., those related to image orthorectification.

A common element of each method is the need to design and measure the GCP point network. This stage of work has a significant impact on the depth accuracy, considering that terrestrial control points should be located on the mainland and that their number must be proportionate to the tested surface size. Exceptions include waterbodies with high water quality. This is why the methods applied are geared towards bathymetric measurements carried out on shallow waterbodies. It should be noted that these methods are based on images taken by UAVs on individual waterbodies. On the basis of the performed analyses, it should be concluded that the most reliable method for determining the shallow waterbody depth is cBathy, as it is the only solution that is repeated in other studies (Depth Inversion and uBathy).

## Figures and Tables

**Table 1:** Geometrical properties for the coral reef on Fuvahmulah Island measurement site.

Length along Shore	Width across Shore	Flight Height	Front Overlap	Side Overlap
140–150 m	70–80 m	25 m	85%	75%

**Table 2:** Measurement locations and equipment according to the method used to determine the waterbody depth.

Method	Location	UAV	Camera
cBathy	Field Research Facility (Duck, NC, USA)	3D Robotics X8+	4x GoPro Hero 4 Black, 4K resolution, 30 fps
Depth Inversion	Mouth of the Oi River in Suruga Bay (Shizuoka, Japan)	DJI Phantom 4	DJI Phantom’s factory camera, 4K resolution, 29.97 fps
SVR	Agia Napa (Agia Napa, Cyprus) and Amathouda (Amathous, Cyprus)	Swinglet CAM	Canon IXUS 220 HS, 4000 × 3000 pixel format
UAV-Derived Bathymetry	Tyrrhenian Sea (San Vincenzo, Italy)	HexaCopter	MAIA WV, sensors: 8 multispectral + 1 RGB, spectrum range: 390–950 nm, 1280 × 960 pixel format
UAV-SfM	Alarm River in Lar National Park (70 km northeast of Tehran, Iran)	Spreading Wings S1000	Canon 5D Mark III, 5760 × 3840 pixel format
uBathy	Victoria Beach (Cádiz, Spain)	DJI Phantom 3 Pro	DJI Phantom’s factory camera, 4096 × 2160 pixel format, 24 fps

**Table 3:** Camera specification, flight height and Ground Sampling Distance (GSD) for Agia Napa and Amathouda measurement sites.

Location	Focal Length	Pixel Size	Pixel Format	Flight Height	GSD
Agia Napa	4.3 mm	1.55 μm	4000 × 3000	209 m	6.3 cm
Amathouda	4.3 mm	1.55 μm	4000 × 3000	103 m	3.3 cm

**Table 4:** Summary of the depth Root Mean Square Error (RMSE) values for the cBathy, Depth Inversion, UAV-Derived Bathymetry (UDB), and uBathy methods. Own study based on [32,33,39,49].

Method			RMSE (m)
cBathy			0.17–0.34
Depth Inversion			0.33–0.52
UAV-Derived Bathymetry	Depth range: 0–5 m	Lyzenga	0.24
		Stumpf	0.37
	Depth range: 0–11 m	Lyzenga	0.89
		Stumpf	1.06
uBathy	Video 1	t_f_ = 0 s	–
		t_f_ = 5 s	0.42–0.73
		t_f_ = 10 s	0.47–0.59
	Video 2	t_f_ = 0 s	0.38–0.44
		t_f_ = 5 s	0.38–0.46

## Data Availability

Not applicable.
